# Evaluation of data completeness in the electronic health record for the purpose of patient recruitment into clinical trials: a retrospective analysis of element presence

**DOI:** 10.1186/1472-6947-13-37

**Published:** 2013-03-21

**Authors:** Felix Köpcke, Benjamin Trinczek, Raphael W Majeed, Björn Schreiweis, Joachim Wenk, Thomas Leusch, Thomas Ganslandt, Christian Ohmann, Björn Bergh, Rainer Röhrig, Martin Dugas, Hans-Ulrich Prokosch

**Affiliations:** 1 Lehrstuhl für Medizinische Informatik, Friedrich-Alexander-Universität Erlangen-Nürnberg, Krankenhausstraße 12, Erlangen, 91054, Germany; 2 Institute of Medical Informatics, University of Münster, Albert-Schweitzer-Campus 1, Gebäude A11, Münster, 48149, Germany; 3 Anaesthesiologie und operative Intensivmedizin, Rudolf-Buchheim-Straße 7, Gießen, 35392, Germany; 4 Universitätsklinikum Heidelberg, Zentrum für Informations- und Medizintechnik, Sektion Medizinische Informationssysteme, Speyerer Straße 4, Heidelberg, D-69115, Germany; 5 Koordinierungszentrum für Klinische Studien, Medizinische Fakultät, Heinrich-Heine-Universität, Moorenstr. 5, Düsseldorf, 40225, Germany; 6 Universitätsklinikum Düsseldorf, Abt. Datenverarbeitung D05.IKT, Anwendungsbetreuung Medico, Moorenstr. 5, Düsseldorf, 40225, Germany; 7 Universitätsklinikum Erlangen, Medizinisches Zentrum für Informations- und Kommunikationstechnik, Krankenhausstraße 12, Erlangen, 91054, Germany

**Keywords:** Patient selection, Research subject recruitment, Clinical trials as topic, Electronic health records, Data quality, Information systems, Database

## Abstract

**Background:**

Computerized clinical trial recruitment support is one promising field for the application of routine care data for clinical research. The primary task here is to compare the eligibility criteria defined in trial protocols with patient data contained in the electronic health record (EHR). To avoid the implementation of different patient definitions in multi-site trials, all participating research sites should use similar patient data from the EHR. Knowledge of the EHR data elements which are commonly available from most EHRs is required to be able to define a common set of criteria. The objective of this research is to determine for five tertiary care providers the extent of available data compared with the eligibility criteria of randomly selected clinical trials.

**Methods:**

Each participating study site selected three clinical trials at random. All eligibility criteria sentences were broken up into independent patient characteristics, which were then assigned to one of the 27 semantic categories for eligibility criteria developed by Luo et al. We report on the fraction of patient characteristics with corresponding structured data elements in the EHR and on the fraction of patients with available data for these elements. The completeness of EHR data for the purpose of patient recruitment is calculated for each semantic group.

**Results:**

351 eligibility criteria from 15 clinical trials contained 706 patient characteristics. In average, 55% of these characteristics could be documented in the EHR. Clinical data was available for 64% of all patients, if corresponding data elements were available. The total completeness of EHR data for recruitment purposes is 35%. The best performing semantic groups were ‘age’ (89%), ‘gender’ (89%), ‘addictive behaviour’ (74%), ‘disease, symptom and sign’ (64%) and ‘organ or tissue status’ (61%). No data was available for 6 semantic groups.

**Conclusions:**

There exists a significant gap in structure and content between data documented during patient care and data required for patient eligibility assessment. Nevertheless, EHR data on age and gender of the patient, as well as selected information on his disease can be complete enough to allow for an effective support of the manual screening process with an intelligent preselection of patients and patient data.

## Background

Together with the growing amount of clinical data collected during patient care, the desire to gain access and to use these data for purposes not related to patient care grows alike. [1] Clinical researchers, quality management, accounting and certification agencies propose a wealth of scenarios to which the supposed knowledge could be applied. [2] One important application of secondary use is the identification of patients for recruitment into clinical trials [3]. The primary task here is to compare the eligibility criteria defined in study protocols with patient data contained in the electronic health record (EHR). Technical challenges arising from the non-structured representation of eligibility criteria within study protocols have been met with successful demonstrations of intermediate formats, such as Arden Syntax [4], ad hoc expressions [5] and Logic-based languages [6]. A comprehensive review can be found by Weng et al. [7] Likewise, systems for automated or semi-automated transformation of eligibility criteria into these computable formats have been developed [6,8].

No EHR can contain patient data on all possible eligibility criteria. Practical applications of systems for recruitment support were thus generally limited to ‘a set of coarse criteria, and on information that is likely to be available in the patient record’ [3]. In current recruitment systems this set of criteria depends on the contents of each local EHR. However, for multi-centre studies a set of common criteria shared by all participating research sites is preferable to ensure that all hospitals include patients with the same characteristics. In order to determine this set of common criteria, the commonly available EHR contents must be taken into account.

A review on the content and quality of EHR data has been presented by Chan, Fowles and Weiner [9]. However, to our knowledge the currently available literature has limitations. While the general content of the EHR has been investigated for primary care practices, for example by Pringle et al. [10] and Scobie et al. [11], investigations regarding the EHR of tertiary care providers have been limited to single health conditions like HIV [12] or pancreatic cancer [13]. Furthermore, all studies need to focus on a selection of data elements, which are defined by the purpose of the investigation. Obviously, an investigation regarding the availability of EHR data for the purpose of patient recruitment should derive its data elements of interest from a random set of real world eligibility criteria. But while the contents of these criteria have recently been described by Luo et al. [14], no comparison with the content of a set of real life EHRs is yet available.

The objective of this research is to determine to what extent the patient data requested in eligibility criteria of clinical trials is available from the EHR of tertiary care providers. The research is conducted by five German university hospitals with different EHR systems.

## Methods

Five German university hospitals located in Münster, Erlangen, Düsseldorf, Heidelberg and Gießen agreed to participate in this research. All hospitals are large tertiary care centres with 1,200 to 1,900 beds. Each site applies another EHR system: Orbis (Agfa) is used in Münster, Soarian Clinicals (Siemens) in Erlangen, i.s.h.med (Siemens) in Heidelberg, Medico (Siemens) in Düsseldorf and a proprietary development named KAOS in Gießen. These systems cover 94% of the EHR products used in all 33 German University Hospitals. All EHR systems offer a single point of access to most of the patient data that is documented during patient care. To achieve this, they import data from a multitude of specialty specific systems like the laboratory, the surgery, the intensive care and the patient management system. It is also possible to enter data directly into the EHR by designing custom assessment forms which consist of a set of data elements like free text and numeric fields, checkboxes and multiple choice questions. The content of the EHR is determined by the individual requirements of the hospital administration and the clinical departments.

Each hospital compared the eligibility criteria of three trials with the patient data available from its local EHR. The set of studies was selected individually for each hospital. Selected trials had to meet the following conditions: (1) The disease under investigation was still treated by the same department that conducted the trial. (2) The permission to process the clinical data of all patients from that department could be obtained (3). The trial was not sponsored by a pharmaceutical company.

In the trial descriptions from clinicaltrials.gov, eligibility criteria are provided in free text sentences. These sentences can be logically and grammatically complex and contain, for example, Boolean or conditional expressions. The original form of these criteria is therefore ill suited for direct comparison with the more structured data elements in the EHR, which usually hold only a single piece of information. As a consequence, we broke up all eligibility criteria sentences into independent patient characteristics. We define a patient characteristic as a single fact that is needed to evaluate an eligibility criterion for a given patient. To assess for example the criterion ‘Lupus nephritis with renal biopsy performed within one year prior to screening’ the two characteristics ‘patient suffers from lupus nephritis’ and ‘date of renal biopsy’ must be known. All logical relations between these characteristics as stated in the criterion’s original free text sentence were discarded in this process.

Clustering of the patient characteristics by content is necessary to allow a meaningful presentation of results. For this reason, all characteristics were manually assigned to one of the 27 semantic categories defined for eligibility criteria by Luo et al. [14] This research group recently used UMLS-based semantic annotation and hierarchical clustering on 4.821 randomly selected eligibility criteria to identify 27 semantic categories in six topic groups. The topic groups are: ‘health status’, ‘lifestyle choice’, ‘treatment or healthcare’, ‘diagnostic or lab result’, ‘demographics’ and ‘ethical consideration’. Five authors each assigned all eligibility criteria of 3 studies to one of the semantic categories. Two authors validated the results. The distribution of the criteria over the semantic categories was compared with the results reported by Luo et al. to assess the representativeness of the given trials.

**Figure 1:**
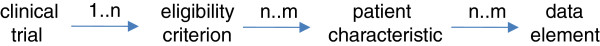
**Terminology used in our study.** Detailed legend: Each trial contains 1 to n eligibility criteria to describe the patient population under investigation. Each criterion contains one to n patient characteristics, which must be known in order to evaluate whether the criterion is true for a given patient. Each patient characteristic can be mapped to 0 to n data elements in the EHR, which hold the relevant data regarding the characteristic.

In the next step, each patient characteristic was matched to its corresponding data elements in the EHR. We defined corresponding data elements of a patient characteristic as those fields in the EHR’s database which hold for at least one patient the information whether the patient has the characteristic or not. Corresponding data elements were identified by (1) individual knowledge of the database administrators, (2) searching for keywords in the EHR metadata (for example in the names of laboratory values or assessment form elements) and (3) involvement of the clinical staff, which actually generates the clinical documentation during patient care. Only numeric and structured element types like checkboxes and drop down menus were included, as none of the participating hospitals had the means to reliably extract information from free text data elements. The terminology used in this paper is summarized in Figure 1.

Completeness of EHR data for the purpose of patient recruitment depends on two conditions. First, data elements need to exist, which enable the physician to document a given patient characteristic. If an EHR lacks the necessary data elements, no data will be available for the characteristic. For each semantic category, we calculated the fraction of documentable patient characteristics as the fraction of patient characteristics with at least one corresponding data element. Second, even if corresponding data elements are offered by the EHR, data will be incomplete, if these data elements are not filled in by the clinicians. We calculated for each patient characteristic the fraction of patients with any data in at least one of its corresponding data elements. The calculation included all patients admitted in the fourth quarter of 2011 to the clinical department that conducted the trial. The results are presented as average values grouped by semantic category. Finally the total completeness of EHR data for the purpose of eligibility determination for clinical trials was calculated by multiplication of the fraction of patient characteristics with corresponding data elements with the fraction of patients with any data in these data elements.

## Results

The 15 trials (see Table 1) comprised at least 3, at most 49 and in total 351 eligibility criteria. Half of the criteria described only one patient characteristic, while the other half required data on 2 to 16 characteristics. After decomposition we obtained 706 patient characteristics. Each trial contained between 11 and 122 patient characteristics. After manual assignment of each patient characteristic to one of the semantic categories we found a quantitative distribution very similar to that described by Luo et al. (see Table 2). Six categories did not appear in our trials: bedtime, exercise, device, receptor status, address and ethnicity. We were not able to relate 22 (3%) of our patient characteristics to the proposed categories, mainly because they did not focus on the patient, but on the cause of a symptom (‘organ dysfunction not explained by any chronic disease’), on the outcome (‘failed conservative therapy’) or on specifics of the treatment or the environment of the patient (‘[method of] contraception results in a failure rate less than 1% per year’). Two thirds of all information needed to assess the eligibility of a patient for a trial were related to his disease history (health status and diagnostic or lab test), while another 16% related to his treatment history (Treatment or Health Care).

**Table 1:** Selection of trials included into our study

**Hospital**	**Study identifier**	**Disease**	**Parent population**	**Number of criteria**
Münster	NCT01177033	intermittent claudication	299	18
Münster	NCT00976222	pigment epithelial detachment	3330	25
Münster	NCT00961142	acute leukemia	2065	39
Erlangen	NCT00866684	skin cancer	10589	21
Erlangen	NCT00025402	chronic myelogenous leukemia	1806	16
Erlangen	NCT00310583	mechanical hyperalgesia	4438	31
Heidelberg	NCT01165671	primary glioblastoma	22280	22
Heidelberg	NCT00176150	anorexia nervosa	22280	3
Heidelberg	NCT00750971	lupus erythematosus	22280	21
Düsseldorf	NCT00798525	critical illnesses	708	13
Düsseldorf	NCT00933374	urothelial carcinoma	2091	40
Düsseldorf	NCT00977132	myelodysplastic syndrome	2351	30
Gießen	DOI 10.1111/j.1365-2044.2012.07303.x	general anaesthesia	5500	7
Gießen	NCT01146821	sepsis	208	49
Gießen	DRKS00003264	abdominal surgery	5500	16

Detailed legend: Each participating hospital selected 3 clinical trials, for which the study identifier, the disease under investigation, as well as the size of the parent population and the number of eligibility criteria are given in this table. Study identifiers beginning with NCT are related to clinicaltrials.gov and the identifier beginning with DRKS relates to the German register germanctr.de. One trial was not registered and thus has no identifier. For this study we show the digital object identifier (DOI) of the publication of the trial’s results. The parent population includes all patients admitted in the fourth quarter of 2011 to the clinical department that conducted the trial.

**Table 2:** Eligibility criteria distribution according to semantic categories

	**Luo et. al.**	**This research**	
	**[%]**	**[%]**	**n**
**Health Status**	**43.72**	**45.04**	**318**
Disease, Symptom and Sign	29.21	22.52	159
Pregnancy-related activity	5.17	5.24	37
Neoplasm status	3.67	3.40	24
Disease stage	2.20	2.27	16
Allergy	2.15	5.95	42
Organ or tissue status	0.73	5.38	38
Life expectancy	0.59	0.28	2
**Treatment or Health Care**	**20.74**	**17.56**	**124**
Pharmaceutical substance or drug	12.84	7.37	52
Therapy or surgery	7.61	10.20	72
Device	0.29	-	0
**Diagnostic or lab test**	**14.85**	**19.41**	**137**
Diagnostic or lab results	14.63	19.41	137
Receptor status	0.22	-	0
**Demographics**	**8.79**	**4.67**	**33**
Age	5.91	2.69	19
Special patient characteristic	1.18	0.42	3
Literacy	0.65	0.28	2
Gender	0.41	1.27	9
Address	0.35	-	0
Ethnicity	0.29	-	0
**Ethical Consideration**	**8.52**	**8.64**	**61**
Consent	2.76	2.55	18
Enrolment in other studies	2.38	1.27	9
Capacity	1.50	3.54	25
Patient preference	1.38	0.57	4
Compliance with protocol	0.50	0.71	5
**Lifestyle Choice**	**3.38**	**1.56**	**11**
Addictive behaviour	2.09	1.42	10
Bedtime	0.47	-	0
Exercise	0.44	-	0
Diet	0.38	0.14	1
**no fitting category**	**-**	**3.12**	**22**

Detailed legend: Distribution of the 706 patient characteristics from 15 clinical trials according to the semantic categories developed by Luo et al. and comparison with the distribution obtained by Luo et al. for 4821 eligibility criteria.

After clustering of all patient characteristics in semantic categories, we subsequently (1) matched these characteristics to corresponding data elements, (2) calculated the fraction of patients with some value for at least one of those data elements and (3) calculated overall data completeness. The results of these three steps are summarized for each topic group. Average values for each semantic group are displayed in detail in Table 3.

**Table 3:** Completeness of patient information in German electronic health records

	**D**	**F**	**C**
**Health Status**	**0.60**	**0.77**	**0.46**
Disease, Symptom and Sign	0.81	0.79	0.64
Pregnancy-related activity	0.16	0.38	0.06
Neoplasm status	0.75	0.79	0.59
Disease stage	0.25	0.45	0.11
Allergy	0.17	0.69	0.12
Organ or tissue status	0.74	0.82	0.61
Life expectancy	0	-	0
**Lifestyle Choice**	**0.82**	**0.82**	**0.67**
Addictive behaviour	0.90	0.82	0.74
Diet	0	-	0
**Treatment or Health Care**	**0.57**	**0.44**	**0.25**
Pharmaceutical substance or drug	0.35	0.17	0.06
Therapy or surgery	0.74	0.46	0.34
**Diagnostic or lab test**	**0.54**	**0.36**	**0.20**
Diagnostic or lab results	0.54	0.36	0.20
**Demographics**	**0.85**	**0.91**	**0.77**
Age	0.95	0.94	0.89
Special patient characteristic	0.33	0.76	0.25
Literacy	0	-	0
Gender	1.00	0.89	0.89
**Ethical Consideration**	**0.08**	**0.71**	**0.06**
Consent	0.06	0.50	0.03
Enrolment in other studies	0	-	0
	Capacity	0.16	0.76	0.12
Patient preference	0	-	0	
Compliance with protocol	0	-	0	
**Total**	**0.55**	**0.64**	**0.35**	

Detailed legend: D = fraction of documentable patient characteristics, i.e. at least one data element containing data on this characteristic was found, F = Average fraction of patients with any data documented in one of these data elements, C = average completeness of patient data for patient characteristics from the semantic category (C=DxF).

### Health status

Corresponding data elements were found for 192 (60%) of 318 patient characteristics in the topic group ‘health status’. In 123 (39%) cases the participating hospitals translated the characteristic into one or more codes from the International Classification of Diseases (ICD). Beyond the ICD catalogue, each hospital had to rely on individual assessment forms. The existence and content of these assessment forms depends heavily on the preferences of each clinical department and thus exhibited a wide variability between the participating hospitals. The semantic categories in the group ‘health status’ are therefore divided into two groups. On the one hand, ‘disease, symptom and sign’, ‘organ or tissue status’ and ‘neoplasm status’ are well covered by the ICD catalogue. Therefore 74 to 81% of the patient characteristics belonging to these categories were found in the EHR and the corresponding data elements were populated for about 80% of all patients. On the other hand, gaps seem to exist in the ICD catalogue for characteristics from the categories ‘allergy’, ‘disease stage’, and ‘pregnancy-related activities’. While corresponding data elements existed in individual hospitals, these were not common to all, resulting in a poor average data completeness of only 6 to 12%. No data elements were found to contain information on the life expectancy of a patient. The overall data completeness in this topic group was 46%.

### Diagnostic or lab test

All studies together requested 137 patient characteristics from the topic group ‘diagnostic or lab test’. The possibility to document the necessary information electronically and in a structured way was given for 74 (54%) of them. Though both were included in one semantic category by Luo et al., diagnostic and laboratory data differed regarding data completeness. Diagnostic data is often measured manually by physicians and nurses. Currently, these results are commonly documented in paper charts rather than in the EHR. In contrast, laboratory and monitoring devices deliver their test results to the physician by electronic means. Thus laboratory data was generally available in a structured format from the EHR. However, challenges arose in identifying data elements corresponding to these patient characteristics, as all five hospitals use individual terminologies rather than LOINC (Logical Observation Identifiers Names and Codes) [15] for data description. Identified data elements for diagnostic and laboratory data were populated for approximately one third of all patients. For this reason, the average data completeness of patient characteristics from this topic group was only 20%.

### Treatment or health care

We assigned 124 patient characteristics to the topic group ‘treatment or health care’. Corresponding data elements were found for 71 (57%) of these characteristics. In 46 (37%) cases, the characteristics were encoded with the ‘Operationen- und Prozedurenschlüssel’ (OPS) catalogue, which is the German modification of the International Classification of Procedures in Medicine (ICPM). Fields from individually designed assessment forms were again chosen as the second data source, where codes were unavailable. Corresponding data elements were identified for 74% of the characteristics from the semantic category ‘therapy or surgery’, but these were in average only populated for half of all patients, resulting in a data completeness of 34%. Structured data on a patient’s medication is currently almost non-existing. Only 35% of the characteristics could be found in the EHR and the corresponding data elements were empty for 83% of the patients.

### Ethical consideration

Only 5 of 61 characteristics in the topic group ‘ethical consideration’ were found for at least one patient in a structured form. This was not due to an insufficiency of the documentation systems but rather to the nature of the required characteristics itself. Data for items from the semantic categories ‘compliance with protocol’ and ‘consent’ are available only after inclusion into the trial. In three cases, questions regarding the capacity of the patient to participate in the trial could be translated to a number of diseases, but more often they were too dependent on the interpretation by the investigator. Finally, while 9 out of 15 trials exclude patients who are enrolled in other trials, this fact is not yet documented in the EHR and thus cannot be taken into consideration. With 6% total data completeness information on characteristics from the topic group ‘ethical consideration’ does virtually not exist.

### Demographics

Characteristics considering age and gender of a patient are available from the EHR for almost every case. No data elements corresponding to the patient’s literacy were found. Three characteristics from the category ‘special patient characteristics’ asked for the patient’s healthiness, his family history and whether or not he was detained. Here, only the one characteristic regarding family history was available from structured data elements.

### Lifestyle choice

10 out of 11 criteria in the topic group ‘lifestyle choice’ regarded a patient’s addictive behaviour i.e. his potential drug or alcohol abuse. Both can be documented as ICD codes which is why both a high possibility to document these characteristics and usage of the corresponding data elements are reported. Often the daily or weekly dose is also documented as free text in assessment forms, but cannot be evaluated automatically by the participating hospitals yet.

In total, the existing EHRs offered an opportunity to document data for 55% of the patient characteristics required to assess the patient’s eligibility for 15 trial protocols. The corresponding data elements were populated for 64% of all patients. Thus the average completeness of patient data was 35%.

## Discussion

Five hospitals analysed the completeness of patient data required for patient recruitment into 15 randomly selected clinical trials. In average, about half of all patient characteristics mentioned in the trials’ eligibility criteria could be documented in structured data elements within their EHR. When a corresponding data element existed it was populated on average with data for two thirds of the patients. While overall completeness of data for patient recruitment was thus only 35%, some semantic categories were more complete than others.

On the one hand, information on the age and gender of a patient is complete for 90% of the patients. Data on the disease, which is currently treated, is complete for 60% of the characteristics and patients. On the other hand, comorbidities and medication are currently only available for about 10% of all patients. Primarily, data elements for billing purposes and laboratory data are available in a structured format. The remaining information generated during patient care is generally captured in paper charts or electronically as free text. The inclusion of the latter for secondary use purposes is likely to improve data completeness, but none of the participating hospitals had the necessary tools to transform free text into structured data.

Eligibility assessment for clinical trials will require patient data to be relatively complete for all observed patients. Unfortunately, the absence of patient conditions is usually not recorded during treatment which leads to missing data. Evaluating patient eligibility based on partly missing data risks missing eligible patients and the introduction of selection errors if the distribution of missing data is not completely random. The decision whether missing data for a specific patient characteristic can be interpreted as absent condition or whether the available data is insufficient for evaluation can only be made individually. It depends on the patient characteristic, the corresponding data elements and how they are used. Therefore, while our results show fractions of missing data of up to 83% (average 36%), this does not necessarily mean that these characteristics cannot be used for eligibility assessment. It does however indicate the risk of introducing selection errors. We believe that manual review and additional documentation will remain necessary for most clinical trials.

Our final result of 35% completeness of data is larger than an estimation made by El Fadly et al. [16] who found only 13% of the data elements required for one trial in their EHR. Other studies are restricted on specific data elements or a limited patient population. The data completeness of 20% for lab results found in our study is similar to that identified by McGinnis et. al. [17] (9 laboratory results, completeness: 1% to 37%, average: 14%) and Persell et. al. [18] (5 laboratory results, completeness: 1.9%, 22.5%, 29.1%, 25.3%, 23.3%). In a review of 4 papers conducted by Thiru et al. [19] in 2003, data completeness for 13 diseases ranged between 40 and 100% with an average of 86%, which is 20% more than our result. The broad definition of the corresponding semantic category ‘disease, symptom and sign’ by Luo et al. might explain this difference.

Luo et al. developed their clusters for entire eligibility criteria sentences on the premise, that ‘each eligibility criterion sentence is an independent patient characteristic’. The eligibility criteria for the 15 studies in our research did in fact consist of several patient characteristics in half of the cases. Nevertheless, assigning these characteristics individually to the semantic groups yielded a distribution very similar to that of Luo et al. While all assignments were checked by at least two of the authors and while most categories were very straightforward, the whole process was manual and thus mismatches cannot be excluded. The number of characteristics in each category, which is given in Table 2, might be a good indicator on how sensitive the results for this category are to mismatching. The average results for all eligibility criteria are not influenced by their distribution.

In an analysis of 1000 random eligibility criteria Ross et al. [20] found 6.8% of the criteria to be incomprehensible, 19% to require clinical judgement and 24% to require additional information beyond that specified in the criterion itself. They conclude that ‘researchers trying to determine patient eligibility for studies face incomprehensible and ambiguous criteria as well as under-specified criteria requiring clinical judgment or assessments.’ Indeed, all five hospitals participating in our study reported difficulties with the mapping of eligibility criteria to data elements contained in the EHR. Codes from the ICD and OPS catalogues were preferred to encode patient characteristics whenever possible. Other terminologies such as SNOMED CT were not utilized by any of the participating hospitals.

Our study is limited to measuring data completeness, which is only one of three fundamental dimensions of data quality identified by Weiskopf and Weng [21]. They define data completeness as the fraction of patients that has some value documented for a given patient characteristic. Additionally, data correctness represents the fraction of available data that is true for the patient and data currency represents the fraction of data that is documented before a specified point in time. Data correctness is the major concern of clinical researchers towards secondary use. [22] The data quality of the most valuable data source identified in our study, billing data in the form of ICD and OPS codes, has already been investigated by many research teams. Even though many sources of errors exist in the course of the coding process [23], a review of 21 studies on coding accuracy in the United Kingdom [24] found the diagnosis codes to be accurate for 96.5% for ICD7, 87% for ICD8 and 77% for ICD9. A very high accuracy of 97% was also found for procedure codes (OPS). Laboratory data can be regarded as correct when it is transferred directly from the laboratory device to the EHR without human intervention. Compared to the amount of missing data in the EHR, we believe that incorrectness of data is of minor influence to the feasibility of concrete secondary use measures.

When beginning this work, we expected electronic support of patient recruitment to follow the commonly presented process of (1) translation of eligibility criteria into an electronic form, (2) comparison of the electronic criteria with existing patient data, (3) presenting the user with a list of patients that (a) fulfil all inclusion criteria and (b) do not fulfil any exclusion criterion. From the experience gained from the data analysis we believe direct translation from the eligibility criteria of a trial is currently not an efficient approach. System developers also need to consider the completeness of EHR data and how it fits the required patient characteristics. Otherwise many patients will not be presented to the investigator due to lack of data, thus risking that the included set of patients is not representative of the target population. In most cases, the selection of EHR data elements will therefore require the involvement of the documenting physicians and nurses. Often, an intelligent presentation of patient data for screening combined with well-placed reminders will be more helpful to the investigator than the attempt to assess the eligibility of patients.

If patient care and research are to interlock more tightly both parties need to improve towards this goal. Electronic documentation of patient history and treatment process is currently still too fragmented for some secondary use purposes. More and more documentation should be captured electronically. In particular, incentives are needed to convince physicians to document more data in a structured form within the EHR. To promote this process medical informatics can function as a catalyst by providing tools and knowledge on how to capture and evaluate data. The greatest challenge hereby is to strike a balance between the physician’s ‘freedom of expression’ and the researcher’s need for structure and standardization. The development of free text processing tools to transform and extract structured data from free text will be an important tool to mediate between both worlds.

## Conclusions

There exists a significant gap in structure and content between data documented during patient care and data required for patient eligibility assessment. Because of the high fraction of missing data, developers of computerized recruitment support systems need to be careful which data elements to include into the screening process. Nevertheless, EHR data on age and gender of the patient, as well as selected information on his disease can be complete enough to allow for an effective support of the manual screening process with an intelligent preselection of patients and patient data.

## Abbreviations

EHR: Electronic Health Record; ICD: International Classification of Diseases; LOINC: Logical Observation Identifiers Names and Codes; OPS: Operationen- und Prozedurenschlüssel.

## Competing interests

The authors declare that they have no competing interests.

## Authors’ contributions

FK contributed to the design of the study, executed the study at the Universitätsklinikum Erlangen and drafted the manuscript. BT coordinated the execution of the study, executed the study at the Universitätsklinikum Münster and helped to draft the manuscript. RWM executed the study at the Universitätsklinikum Gießen. BS executed the study at the Universitätsklinikum Heidelberg and helped to draft the manuscript. JW executed the study at the Universitätsklinikum Düsseldorf. TL executed the study at the Universitätsklinikum Düsseldorf. TG executed the study at the Universitätsklinikum Erlangen. CO contributed to the design of the study. BB contributed to the design of the study. RR contributed to the design of the study. MD designed the study and contributed to the manuscript. HUP designed the study and contributed to the manuscript. All authors reviewed and approved the final manuscript.

## Pre-publication history

The pre-publication history for this paper can be accessed here:

http://www.biomedcentral.com/1472-6947/13/37/prepub

## References

[B1] Prokosch HU Ganslandt T Perspectives for medical informatics. Reusing the electronic medical record for clinical research. Methods Inf Med 2009 48 1 38 44 19151882

[B2] Safran C Bloomrosen M Hammond WE Labkoff S Markel-Fox S Tang PC Detmer DE Toward a National Framework for the Secondary Use of Health Data: An American Medical Informatics Association White Paper J Am Med Inform Assoc 2007 14 1 9 17077452 10.1197/jamia.M2273 PMC2329823

[B3] Cuggia M Besana P Glasspool D Comparing semi-automatic systems for recruitment of patients to clinical trials Int J Med Inform 2011 80 6 371 388 10.1016/j.ijmedinf.2011.02.003 21459664

[B4] Ohno-Machado L Wang S Mar P Boxwala A Lorenzi NM Decision support for clinical trial eligibility determination in breast cancer Proceedings of the AMIA symposium 6-10 November 1999 Washington, DC, USA 1999 Philadelphia, PA, USA: Hanley & Belfus 340 344 PMC2232554 10566377

[B5] Dugas M Lange M Berdel WE Müller-Tidow C Workflow to improve patient recruitment for clinical trials within hospital information systems - a case-study Trials 2008 9 2 10.1186/1745-6215-9-2 18186949 PMC2253503

[B6] Patel C Cimino JJ Dolby J Fokoue A Kalyanpur A Kershenbaum A Ma L Schonberg E Srinivas K Aberer K, Choi KS, Noy N, Allemang D, Lee KI, Nixon L, Golbeck J, Mika P, Maynard D, Mizoguchi R, Schreiber G, Cudré-Mauroux P Matching patient records to clinical trials using ontologies ISWC'07/ASWC'07 Proceedings of the 6th international semantic web conference and 2nd Asian semantic web conference 11-15 November, 2007 Busan, Korea 2007 Berlin, Germany: Springer-Verlag 816 829

[B7] Weng C Tu SW Sim I Richesson R Formal representation of eligibility criteria: a literature review J Biomed Inform 2010 43 3 451 467 10.1016/j.jbi.2009.12.004 20034594 PMC2878905

[B8] Lonsdale D Tustison C Parker C Embley DW Kop C, Fliedl G, Mayr HC, Métais E Formulating Queries for Assessing Clinical Trial Eligibility NLDB'06 Proceedings of the 11th international conference on Applications of Natural Language to Information Systems 31 May – 2 June, 2006 Klagenfurt, Austria 2006 Berlin, Germany: Springer-Verlag 82 93

[B9] Chan KS Fowles JB Weiner JP Electronic health records and the reliability and validity of quality measures: a review of the literature Med Care Res Rev 2010 67 5 503 527 Epub 2010 Feb 11 10.1177/1077558709359007 20150441

[B10] Pringle M Ward P Chilvers C Assessment of the completeness and accuracy of computer medical records in four practices committed to recording data on computer Br J Gen Pract 1995 45 399 537 541 7492423 PMC1239405

[B11] Scobie S Basnett I McCartney P Can general practice data be used for needs assessment and health care planning in an inner-London district? J Public Health Med 1995 17 4 475 483 8639350

[B12] Forster M Bailey C Brinkhof MW Graber C Boulle A Spohr M Balestre E May M Keiser O Jahn A Egger M ART-LINC collaboration of International Epidemiological Databases to Evaluate AIDS: **Electronic medical record systems, data quality and loss to follow-up: survey of antiretroviral therapy programmes in resource-limited settings** Bull World Health Organ 2008 86 12 939 947 10.2471/BLT.07.049908 19142294 PMC2649575

[B13] Botsis T Hartvigsen G Chen F Weng C Secondary Use of EHR: Data Quality Issues and Informatics Opportunities AMIA Summits Transl Sci Proc 2010 2010 1 5 21347133 PMC3041534

[B14] Luo Z Yetisgen-Yildiz M Weng C Dynamic categorization of clinical research eligibility criteria by hierarchical clustering J Biomed Inform 2011 44 6 927 935 Epub 2011 Jun 12 10.1016/j.jbi.2011.06.001 21689783 PMC3183114

[B15] Forrey AW McDonald CJ DeMoor G Huff SM Leavelle D Leland D Fiers T Charles L Griffin B Stalling F Tullis A Hutchins K Baenziger J Logical Observation Identifiers, Names, and Codes (LOINC) database: a public use set of codes and names for electronic reporting of clinical laboratory results Clin Chem 1996 42 81 90 8565239

[B16] El Fadly A Rance B Lucas N Mead C Chatellier G Lastic PY Jaulent MC Daniel C Integrating clinical research with the Healthcare Enterprise: from the RE-USE project to the EHR4CR platform J Biomed Inform 2011 44 1 94 102 Epub 2011 Aug 25 10.1016/j.jbi.2010.10.002 21888989

[B17] McGinnis KA Skanderson M Levin FL Brandt C Erdos J Justice AC Comparison of two VA laboratory data repositories indicates that missing data vary despite originating from the same source Medical Care 2009 47 121 124 10.1097/MLR.0b013e31817d69c2 19106740 PMC3032537

[B18] Persell SD Dunne AP Lloyd-Jones DM Baker DW Electronic health record-based cardiac risk assessment and identification of unmet preventive needs Medical Care 2009 47 418 424 10.1097/MLR.0b013e31818dce21 19238100

[B19] Thiru K Hassey A Sullivan F Systematic review of scope and quality of electronic patient record data in primary care BMJ 2003 326 7398 1070 10.1136/bmj.326.7398.1070 12750210 PMC155692

[B20] Ross J Tu S Carini S Sim I Analysis of eligibility criteria complexity in clinical trials AMIA Summits Transl Sci Proc March 12–13, 2010 2010 Bethesda, MD: American Medical Informatics Association 46 50 PMC3041539 21347148

[B21] Weiskopf NG Weng C Methods and dimensions of electronic health record data quality assessment: enabling reuse for clinical research J Am Med Inform Assoc 2013 20 144 151 10.1136/amiajnl-2011-000681 22733976 PMC3555312

[B22] van der Lei J Use and abuse of computer-stored medical records Methods Inf Med 1991 30 79 80 1857252

[B23] O'Malley KJ Cook KF Price MD Raiford Wildes K Hurdle JF Ashton CM Measuring Diagnoses: ICD Code Accuracy Health Serv Res 2005 40 5 Pt 2 1620 1639 16178999 10.1111/j.1475-6773.2005.00444.x PMC1361216

[B24] Campbell SE Campbell MK Grimshaw JM Walker AE A systematic review of discharge coding accuracy J Public Health Med 2001 23 3 205 211 10.1093/pubmed/23.3.205 11585193

